# Book Reviews

**Published:** 1804-11-01

**Authors:** 


### CRITICAL ANALYSIS
### OF THE
### RECENT PUBLICATIONS
### ON THE
### DIFFERENT BRANCHES OF PHYSIC, SURGERY, AND MEDICAL PHILOSOPHY.

*Schola Medicinæ, or the new Universal History and School of Medicine; translated into English from the original Edition,* by W. Rowley, M. D. &c. &c. 1 vol. 4to. pp. 314. London 1803.

This extensive work, which cost the industrious author the principal part of his medical leisure during the last twenty years, contains the following parts or subjects, viz. Introduction, History of Medicine with Errors of Medical Sects, Osteology, Myology, Angiology, Neurology, Splanchnology, and a Compendium of Physiology, Pathology, and Symptomatology.

The *Introduction* contains a brief Synopsis of the original Edition, and a general explanation of the plates, which are numerous, and well calculated to elucidate the different subjects of the work.

In the history of medicine, Dr. R. appears to have paid great attention to the opinions and practice of Hippocrates, which he details under accounts of his anatomical knowledge, his theory of generation, his medical practice, his materia medica, the diseases of women and children, his midwifery, and, lastly, the surgery of Hippocrates, for at this time all these branches were professed by the same person. As we think a short account of this great man's *practice* in medicine will gratify many of our readers, we shall subjoin it. "In general, his practice is to keep his patients on the water gruel plan: to wait patiently, and watch diligently, for whatever course nature may take to expel the disease, in which salutary purpose he assists. Although his most common remedies are emulsions, hydromels, thin farinaceous ptisans and oxymels; yet, when necessary, he employs bleeding, purging, vomiting, and sweating."

The following Abstract will shew his use of particular remedies, as well as the state of the materia medica in his time.

"BARLEY WATER, HYDROMEL, AND OXYMEL.—In acute diseases, and in epidemic fevers.

CASTOR AND MYRRH.—For the diseases of women, obstructions, and hysteric affections.

ACETUM.—For sore throats, ardent fevers, vomitings, phrenzy, peripneumony, pleurisy, inflammations, and viscidites.

GARLICK.—For cold phlegm, and inflammations of the lungs.

ALLUM.—To cure hæmorrhages, check uterine discharges, and strengthen the uterus, &c.

SPICES.—To promote the menses, and cure phlegmatic diseases.

FRESH OX GALL.—As a laxative to kill worms, for purging suppositaries, and uterine pessaries.

CANTHARIDES.—In dropsies, and to promote the urine, and menses.

DIET OF ONIONS.—For the jaundice, and to promote conception.

LONG ABSTINENCE FROM FOOD.—In dropsies, jaundice, diarrhœas, gouty, or rheumatic pains, asthmas, and disorders of the lungs and spleen.

CLYSTERS.—For pains and overfulness in the head, dry, hot, and windy cholics, pains of the womb, abdomen, pleurisy, fevers, pains of the loins, &c.

CUPPING.—For pains in the head and eyes, bruises, peripneumony, pains of the hip, and other parts.

ELATERIUM.—To purge bile, expel the fœtus, or purge in cancers, ulcers, jaundice, sore throat, &c.

FRICTIONS.—With oil, to strengthen weak joints, and relax stiff ones.

COLD BATH.—For faintings and hysterical fits, to restrain the menses, prevent miscarriages, rheumatic pains.

TO BE AVOIDED.—In diseases of the lungs, as asthmas, coughs, consumptions, &c. diseases of the liver, and tabes dorsalis.

JUNIPER BERRIES.—As a powerful diuretic, to provoke the discharge of urine.

ASSES MILK.—In excessive fluxes from the bowels or womb, for slow fevers, consumptions, and diseases of the lungs.

LINSEED.—In wounds and ulcers, and outwardly in emollient anodyne fomentations.

SOUR APPLES.—To be made into drink for fevers.

MECONIUM OR POPPIES.—For excessive fluxes, and pains in the uterus.

HONEY.—For fevers and inflammations as a resolvent. As a pectoral in coughs, and a laxative in clysters.

MINT.—A stomachic and cordial for vomiting, jaundice, and weak stomachs.

MYRRH.—For most disorders of the stomach, for obstructions of the menses, and to cleanse ulcerations in the mouth and gums.

NITRE FROM EGYPT, MORE LIXIVIOUS THAN OURS, OF A RED COLOUR. DIASCORIDES.—For sore throats, pleurisies, gouty and rheumatic pains, to purge phlegm from the bowels, water in an anasarca, for the schirrus womb, and indurations in general.

Origanum or thyme.—For cold phlegm, dropsies, jaundice, and all sluggish indolent diseases.

Eggs.—Their whites to be given in fevers not ardent, in the drinks, and their yolks for coughs in children, excessive uterine fluxes, and all weaknesses, or relaxations.

Poppy juice.—For hysteric pains, and convulsive disorders, hectic fevers, diarrhœa, and dysentery.

Tar.—Inwardly for ulcers, to expel water from the womb.

Pepper.—Outwardly for the tooth ach, and for convulsions, or cramps.

Cerus of lead.—For disorders of the eyes, skin, and sharp ulcerations.

Penny royal.—For fevers and hysterical diseases, and the diseases of women in general.

Galbanum.—Recommended as an expectorant and promoter of uterine discharges.

Rezin of turpentine.—For inward ulcers, and excessive fluxes, and uterine diseases.

Rose leaves.—For a diarrhœa, diabetes, and relaxation of the uterus, fluor albus, &c.

Elder berries.—To purge in dropsies and uterine diseases.

Scammony root and juice.—To purge in the sciatica, nephritic complaints, and chronic diseases.

Squills.—To purge in uterine complaints, and to be taken in consumptive cases.

Tapping.—For a dropsy and *empyema*.

Whey drink.—For the cure of ulcerations, consumptions, fever, and the gout.

Assa fœtida.—For hysterics, peripneumony, pleurisy, jaundice, and a very large dose to purge bile.

Sulphur.—For ulcers, diseases of the lungs, and *cutaneous* disorders.

Frankincense.—For ulcerations, puerile asthmas, stomachic, and uterine complaints.

It does not appear, that Hippocrates gave powerful narcotics, to procure sleep; though in some few passages of his book, of the disorders of women, he speaks of the juice of poppy, as conducive to the cure of what we now call hysterics. He likewise takes notice of mandrake, but cautions against giving it in quantities, sufficient to cause madness; and he mentions much the same of henbane.

As to baths, suffumigations, fomentations, incissions, and gargarisms, he seems to have been perfectly well acquainted with their efficacy, and the proper seasons and manner of using them. He lays a particular stress upon ointments, but no where mentions plasters. Instead of these he frequently recommended cataplasms, in cases where, even we, perhaps, might find them preferable to plasters.

When bleeding, and the use of purgatives, which were his general means for diminishing the superfluity of blood, or humours, were not sufficient, he then had recourse to diuretics. This he seems to insinuate in his work De Ratione Vict. in Acutis. All diseases terminate, or are cured by evacuations, made either by the mouth, belly, the bladder, or some other outlet; but sweat is common to all diseases, and equally terminates all. For these purposes, he sometimes ordered a bath, at other times sweet wine, garlic, onions, leeks, cucumbers, melon, citruls, cysticus, both sorts of apium, fennel, maidenhair, and night shade, as well as all acid substances. These several remedies he directed in various chronical disorders, after purgation, when he believed the blood to be still loaded with ichor. In some cases he excited a diaphoresis, but does not inform us how he produced it."

"His sentiments of the manners of a physician are worthy of attention. He says, he ought to dress decently, to be grave in his manners, moderate in his actions, chaste and modest in the conversation he is obliged to have with women; no idler, ready to answer every body with candour, sober, patient, always ready to do his duty, without disturbing himself; and he thought it requisite, for the credit of the physician, that he should have a *healthful look*, and a good complexion; for men are apt to suspect him who has not his own health, to be scarcely instrumental to procure another's.

But what he is justly entitled to admiration for, by practitioners in medicine, is, his generous acknowledgement of his mistakes, and ill success. A remarkable instance we find recorded in the fifth book of his Epidemics. For being called to Antonomous, who had received a wound in his head, he unfortunately mistook the wound for one of the sutures, and neglected trepanning him. Some days after, the patient being seized with a great pain in his side, and convulsions in both arms, he was sensible of his error, and tried the trepan, but in vain; for it being the fifteenth day, and the summer season, the patient died the next day.

This candid declaration of his ignorance being the cause of a patient's death, must be admired in all ages; but how few follow his steps in this particular! how eager we are for publishing our success, and how silently we draw a veil over our blunders! This great author desired of the gods, in recompence of his labour, neither riches, nor pleasure; but a long life in perfect health, success in his art, and to render himself famous to posterity. This desire of his, is declared in his oath; and it was accomplished in its full extent; for he lived one hundred and nine years, in soundness of mind and body. He succeeded so well in his art, that he has ever been regarded as the founder of it. He is to physicians, what Homer and Demosthenes are to poets and orators. He received, during his life, such great honours, as were never bestowed on any mortal. The Argians erected a statue of gold to his honour, and the Athenians decreed a crown of the same metal; passed an act, that himself and descendants should be maintained in the Prytaneum; and they initiated him into the great mysteries, an honour rarely conferred on strangers, and never before on any but

Hercules;

Hercules; and he has left behind him, in his works, an immortal reputation; for he has been always considered the original interpreter of Nature; and it is highly probable he will ever preserve his glory, which above two thousand years have not yet robbed him of. And though, even now, some designing professors make a point of obscuring the brightness of his fame, by unmeaning sneers, and dark insinuations; yet we are of opinion, that our ancient author will revive, and receive additional lustre, when the works of such men perish, and are lost in that oblivion they jusly merit. This excellent man died in Thessaly, in the second year of the hundred and seventieth olympiad, three hundred and forty-nine years before the birth of Christ, and was buried between Larissa and Gortona."

( To be continued. )

---

*Experiments proving Vacciolation, or Cow-Pox Inoculation, to be a permanent Security against Small-Pox; with Facts and Remarks. By* Samuel Hill, *Surgeon, Town of Portsea, and Surgeon in the Royal Navy.* 8vo. pp. 47. Portsea, 1804.

It must afford peculiar satisfaction to the advocates for the Jennerian Inoculation, that in that quarter where its failure was supposed to have been detected, the most lucid proofs of its efficacy, when carefully administered, have been exhibited. In this pamphlet, dedicated to the President and Members of the Royal Jennerian Society, the cases, perspicuously detailed, very completely establish the position of the title.

"Vacciolation," says the author, "has been found to be, beyond dispute, a permanent prophylactic against variolous infection; the immense mass of evidence, collected in England alone, and laid before a committee of the House of Commons, by the first medical characters and other men of science, in the United Kingdom, and upon which that committee decided, is sufficient to stamp its value without the aid of foreign testimonies. It may however be remarked, that it is now practised in most parts of the known world, with an astonishing success: in short, in all the quarters of the globe, respectable medical men, as well as other philanthropists, are humanely extending its benefits to thousands; many of whom might otherwise fall victims to the greatest enemy of the human race, the small-pox.

"I commenced the new practice December 5, 1800, and from that to the present period have vacciolated two hundred and thirty, not one of which number has ever taken the casual small-pox, though exposed to its effluvia in all possible ways; many of them having been in contact repeatedly, and even put into the same bed with those who had the confluent small-pox so bad as not to survive that dreadful and truly loathsome disease."

"To extreme care in the choice of vacciolous matter, and particularly in vacciolation, with an attentive observance of the progress of the vesicle, areola, &c." Mr. Hill attributes the success he has had. "At all times when in my power, I had the subjects to be be vacciolated carried to the houses where those resided, from whom I was to take vacciolous matter; and this always on the eighth day or early on the ninth from vacciolation; I do not recollect ever using matter taken before the former period or after the latter."

Considering the promptness with which some gentlemen resort to experiments, with doubtful matter, on subjects not yet protected, we cannot withhold the following accounts; nor refrain from observing, that the last case of the author's is unhappily not the only one on record of 'wisdom at one entrance quite shut out' by the variolous inoculation.

"Before I proceed to relate the experiments, I will beg leave to mention some unfortunate cases of small-pox, which I have witnessed in the course of my practice.—In 1797, I was desired to visit a female child in St. James's Street, Portsea, who had the casual small-pox of the confluent kind, very full; and she was altogether so ill as to allow me to pronounce a very doubtful prognostic. The parents informed me that there was a pustule on the left eye, on which account *only*, they wished my advice. On examination the seventh day from the first appearance of the eruption, I discovered a pustule, fully maturated, on the pupil: I told them, that if the child escaped with life, she would certainly lose the eye; as I conceived it had (the pupil) already suppurated: they said, that if I could not promise to preserve the sight, I need not take the trouble of repeating my visit; but, in the course of eight hours afterwards, they again sent for me in haste, and shewed me the remains of the pupil on a piece of paper, which had been forced out of the orbit in a fit of coughing. This child escaped with life: the tunica albuginea, seemed, after a time, to fill up the vacuum occasioned by the loss of the pupil and iris, which last had also suppurated: the child had a most ghastly appearance.

"I was desired to visit a child of Mr. Palmer, of Hanover-Street, Portsea, in 1799, aged ten years: I found her with symptoms of fever, which ran so high, and the head was so much affected, that I apprehended she would not live till morning, if she was not relieved by an eruption. Some blood was taken from the arm, and the bowels opened by an aperient cathartic, and she was put into the warm bath; the day after, July 7, eruptions appeared, which soon proved to be small-pox. The feverish symptoms now abated, and the head, comparatively speaking, was well. About two hundred pustules maturated, three or four of which came on the pupil of the left eye, which occasioned the loss of it.

"The daughter of Mr. Harfield, then about eighteen months old, was taken ill in the summer of 1803, with feverish symptoms, which proved to be small-pox. I was asked to see her on the eighth day of the eruption; a pustule appeared on one of the eyes; the child had the disease very light, but had the appearance of violent ophthalmia. Every thing was done to moderate the local inflammation, which was treated the same as if the small-pox had been out of the question; but without obtaining the desired end; the the pupil suppurated, and was discharged in the shape of pus. This poor child is now living, and whenever I see her, I lament that she had not been previously vacciolated.

"I was desired to see the infant daughter of Mr. Bruce, Half-way-houses, Portsea, the seventh day after the small-pox appeared. She had been inoculated by a woman, and over the whole surface of the skin I could not reckon more than thirty pustules; and very unfortunately one of those came on the pupil of one of the eyes; the loss of the sight of which was the consequence. To these unfortunate cases, a long catalogue may be added, exclusively of those who have died of the small-pox. In the course of my diurnal visits to different parts of the island of Portsea, I frequently meet some of the children who are subjects of the preceding cases, which never fail to bring to my mind the unbounded goodness of the Deity, in furnishing an antidote to this pestilential disease, through the great and truly philanthropic Dr. Jenner. Contrasting the mildest state of variolation, or small-pox inoculation, with vacciolation, there is a great balance of good, in favour of the latter, which neither occasions death nor loss of sight; nor does it produce scrophula, or any other complaint likely to render life unpleasant: and if I here allow, for argument's sake, (for on no other principle can I allow it) that the cases lately brought forward as failures, are really so; considering the little inconvenience which attended the subjects of them, and the few eruptions which were produced, still it would not make against the general practice of vacciolation: for I beg leave to ask, where is the fond parent who would not with extatic delight court vacciolation, for his or her, perhaps, only child, to ensure so mild a kind of small-pox, and thereby escape all the horrors and deformities of those children, whose cases I have just related? I would add, that in my opinion, if all the cow-pock cases, in these towns, from 1800 to the present time, were failures, they could not make much against the new practice—it would, comparing these towns, to all others where it has been crowned with such astonishing success, appear but as a single drop of water compared with the ocean, or as an atom of matter to the globe itself."

After this exhibition of some of the dire effects of small pox, which fell under the author's immediate notice, he gives some appropriate extracts from Dr. Tytler's translation of the Pædotrophia of Scevole de St. Marthe.

The ten experiments instituted by Mr. Hill between three and four years after vacciolation, had nearly the same results as those of Mr. Creighton and the other gentlemen we have mentioned in our late Numbers.

"In all the preceding experiments, it is remarkable that very early inflammation took place; and that the punctured part rose above the surface of the skin in twenty-four hours after the insertion of the matter. In Mr. Gain's child, as early as twelve hours from variolation, inflammation and elevation had both taken place. Itching was more or less troublesome in all the experiments: and much more so than ever I saw in small pox inoculation.

tion. This early inflammation served to confirm me in my opinion, that their habits were impervious to variolous matter in the way of inoculation; and their resisting the casual small pox, certainly proves that they were rendered insusceptible of it, by the previous vacciolation. Nature, by promptly assembling her forces, at the very point where the enemy had assailed her, shewed that she was determined he should not enter her dominions: she therefore wisely carried on the contest at a distance from the capital, and the enemy experienced a defeat at the very place where he had hoped to gain a victory.

"The inflammation and punctures in all these experiments were of a darker colour, and had a harder feel, than in common small pox inoculation; the hardness was always longer going off than either inflammation or eschar. I have no doubt that I could, with lymph from the punctures, have given the small pox to any one susceptible of that disease.

"Having now completed these experiments, I shall hereafter hold it imprudent to variolate after vacciolation; and I shall decline in future putting my young patients to that test, except at the particular desire of parents; for it has been proved by experiments heretofore, as well as lately made, that morbific matter, and particularly the variolous, cannot always be introduced between the cuticle and cutis with impunity. If vacciolated persons will resist the casual small pox, which there can be no doubt of, it is quite sufficient.

"I will relate a case which occurred in 1801, which greatly tends to recommend the general practice of vacciolation, and particularly under similar circumstances.

"I was called to a poor woman in Havant-street, named Perkins, who had that same day only arrived from Plymouth in one of his Majesty's frigates, on board of which her husband served in the quality of a quarter-gunner. The poor creature fell in labour in the course of four hours after she took possession of her lodgings, and of course no accoucheur had been provided; nor indeed any preparation made for the event; in less than an hour the infant was born. Having retired into another apartment, I was much hurt on re-entering the bed-chamber half an hour after, to find her in tears. Upon enquiring what the cause was, she said that she was no sooner out of one trouble than she had fallen into another, for a child was lying dead in the next room, and another extremely ill, both of the small pox. She then asked me to inoculate her infant from the surviving child; it had the confluent small pox very full indeed, and being the month of July and very warm, I told her I thought she had better not think of it. I then mentioned cow-pock inoculation, and recommended it as likely to preserve the life of her infant; she consented, and it was immediately vacciolated (with matter taken from Mr. Purver's child the preceding month) before it was an hour old. It went through the progress with the greatest regularity; the eschar did not fall off till more than five weeks from vacciolation, and a beautiful characteristic mark was left on the arm.

MEDICAL

